# Genetic connectivity between land and sea: the case of the beachflea *Orchestia montagui* (Crustacea, Amphipoda, Talitridae) in the Mediterranean Sea

**DOI:** 10.1186/1742-9994-10-21

**Published:** 2013-04-25

**Authors:** Laura Pavesi, Ralph Tiedemann, Elvira De Matthaeis, Valerio Ketmaier

**Affiliations:** 1 Unit of Evolutionary Biology/Systematic Zoology, Institute of Biochemistry and Biology, University of Potsdam, Potsdam, D–14476, Germany; 2 Department of Biology and Biotechnology “Charles Darwin”, University of Rome “Sapienza”, Viale dell’ Università 32, Rome, 00185, Italy

**Keywords:** *Orchestia montagui*, Talitrids, Mediterranean Sea, Phylogeography, Mitochondrial DNA, Microsatellites, Allozymes, Approximate Bayesian Computation

## Abstract

**Introduction:**

We examined patterns of genetic divergence in 26 Mediterranean populations of the semi-terrestrial beachflea *Orchestia montagui* using mitochondrial (cytochrome oxidase subunit I), microsatellite (eight loci) and allozymic data. The species typically forms large populations within heaps of dead seagrass leaves stranded on beaches at the waterfront. We adopted a hierarchical geographic sampling to unravel population structure in a species living at the sea-land transition and, hence, likely subjected to dramatically contrasting forces.

**Results:**

Mitochondrial DNA showed historical phylogeographic breaks among Adriatic, Ionian and the remaining basins (Tyrrhenian, Western and Eastern Mediterranean Sea) likely caused by the geological and climatic changes of the Pleistocene. Microsatellites (and to a lesser extent allozymes) detected a further subdivision between and within the Western Mediterranean and the Tyrrhenian Sea due to present-day processes. A pattern of isolation by distance was not detected in any of the analyzed data set.

**Conclusions:**

We conclude that the population structure of *O. montagui* is the result of the interplay of two contrasting forces that act on the species population genetic structure. On one hand, the species semi-terrestrial life style would tend to determine the onset of local differences. On the other hand, these differences are partially counter-balanced by passive movements of migrants via rafting on heaps of dead seagrass leaves across sites by sea surface currents. Approximate Bayesian Computations support dispersal at sea as prevalent over terrestrial regionalism.

## Introduction

In many marine species genetic structure is often shallow as a consequence of large population sizes coupled with high potential for long distance dispersal. A high number of polymorphic markers, large sample sizes and *ad-hoc* designed sampling schemes are hence necessary to unveil population structure [1]. On the other hand, phylogeographic patterns with a clear geographic component are relatively common and easy to detect in terrestrial and/or freshwater species due to the intimately fragmented nature of their habitats [2]. Phylogeographic patterns in species living in the transition area between sea and land (supralittoral zone) have received relatively little attention compared to the wealth of work done on marine and terrestrial species [3-6]. However, a proper understanding of the evolutionary trajectories governing coastal communities is fundamental, especially in light of the increasing pressure that human beings have been exerting on them in the last decades [7].

Talitrid amphipods are often the dominant animal group in the supralittoral zone, where they are confined to a narrow area avoiding both the arid environment inland and submersion in seawater. Active dispersal is restricted to short, mostly nocturnal, foraging movements along the beach [8,9]; maximal active distance recorded for talitrids in nature is 200 m [10]. Talitrids lack any planktonic larval stage and consequently they cannot rely on this stage for long-distance passive dispersal. They are, however, considered as facultative rafters [11,12]. Generally speaking, rafting involves the traveling of organisms over the sea surface via floating material. Talitrids are often associated to wrack, wood or other floating material that could be washed away by waves and transported by sea surface waters from one beach to another. Rafters can be transported from short to medium-long distances and arrive in otherwise unreachable areas [12]. This process has a special impact on those species that are not able to actively disperse over long distances. In particular, it could potentially increase connectivity among populations, thus reducing the onset of local genetic divergence. Peracarid crustaceans are found in both high diversity and abundance on floating items, which seemingly play an important role in their life histories [12].

The semi-enclosed Mediterranean Sea is surrounded by thousands of kilometers of coasts. Previous genetic studies focused on multiple Mediterranean populations of seven different species of talitrids [8,13-18] have revealed two main patterns of genetic structuring, corresponding to two groups of species with contrasting life histories. Those species (i.e. *Orchestia montagui*, *O. mediterranea*, *Platorchestia platensis*) that live within beached decaying seagrass, wrack or gravel, showed a shallow genetic structuring even on a wide geographical scale. Species (i.e. *Talitrus saltator*, *O. stephenseni*, *Macarorchestia remyi*) that are exclusively associated to sandy beaches or stranded rotting logs and have the tendency to burrow into the substrate were invariably highly structured genetically [17,18]. De Matthaeis et al. [8,15] interpreted these alternate patterns in terms of probabilities for talitrids to realize passive dispersal via rafting by being dragged away by waves. These probabilities would be high for the species belonging to the first ecological group because they colonize ephemeral habitats close to the waterline. On the other hand, they would be low for species of the second group bound to sand beaches or rotting logs; these species colonize the upper supralittoral zone and are not well suited to survive on rafts [19].

**Figure 1:**
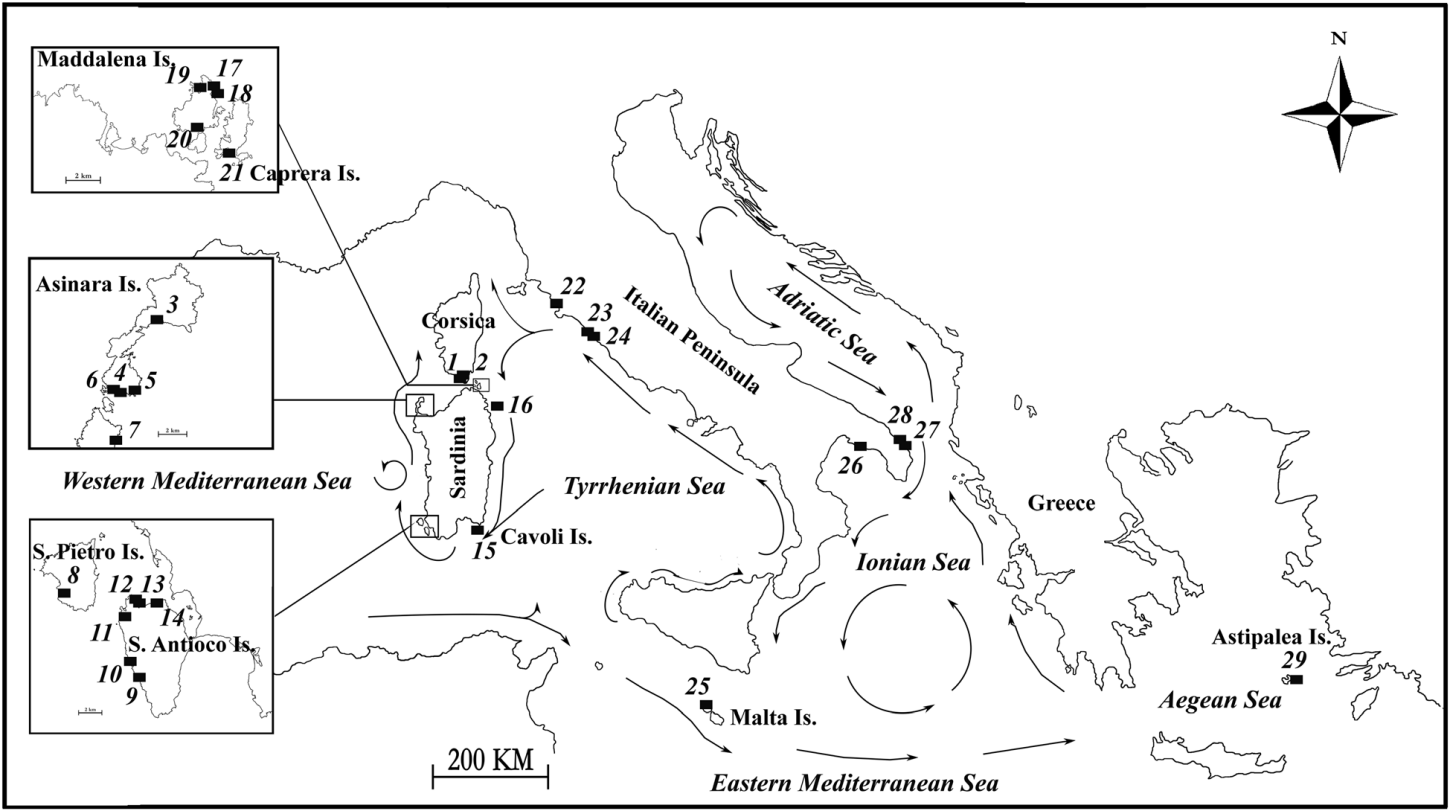
**Geographic distribution of sampling sites for the *O. montagui *populations included in the study.** Details on each sampling site are given in Table 1. The names of the six sampled Mediterranean sub-basins are indicated along with the pattern of the main surface sea currents.

Here we focus on *Orchestia montagui* Audouin 1826 and we expand the data available on the species both in terms of geographic sampling and kind of molecular markers used. The species is abundant along the coasts of the Mediterranean Sea and reaches the Black Sea on the East ([8,20]; Pavesi pers. obs.); it is commonly found in large numbers within banks of beached dead leaves of *Posidonia oceanica*, a seagrass endemic to the area. A number of previous studies, all based on allozymes, considered mostly populations from islets surrounding the island of Sardinia (Western Mediterranean and Tyrrhenian Sea), where *O. montagui* is the most common talitrid, and a few populations from the Eastern Mediterranean and Aegean Sea [8,9,13,21]. At that geographic scale and for allozymes, the species resulted poorly structured. We recently developed eight highly polymorphic microsatellite loci for *O. montagui*[22]. We used these loci, together with a fragment of the mitochondrial DNA (mtDNA) coding for the cytochrome oxidase subunit I (COI), to coarsely assess relationships among six *O. montagui* populations from five Mediterranean basins (Tyrrhenian, Ionian, Adriatic, Western and Eastern) [23]. In that study both classes of markers congruently retrieved a major phylogeographic break between Ionian and Adriatic populations on one side and Tyrrhenian, Western and Eastern Mediterranean populations on the other side, thus capturing a pattern of divergence that escaped allozymes. These results suggested that the lack of structure initially found with allozymes was probably due to their relatively poor resolving power. We planned the present study to overcome the sampling limitations of [23] and to place for the first time under the same analytical frame previous allozyme data along with new mtDNA and microsatellite information collected for the largest number of sampling localities we could access along the coasts of the Mediterranean Sea. We were able to expand our samplings to a total of 26 populations, and thus attain a more detailed picture of patterns of genetic connectivity in *O. montagui*. These populations were sampled in six different Mediterranean basins (see details in Table 1 and Figure 1) and screened for variation at mtDNA and microsatellites. To contrast genetic divergence at different geographical scales, we included in the study populations that were separated from a very few to hundreds of kilometers. In order to make possible combining the data acquired for this study with those previously published in De Matthaeis et al. [8] we re-sampled in thirteen out of the sixteen locations included in that paper.

**Table 1:** List of populations, genetic indices and different localities/samplings included in the study

**Pop**	**Sampling location**	**Code**	**Basins**	**N (COI\msat\alz)**	**Hapl**	**H**	***Π***	***π***_***n***_	**H**_**E**_	**H**_**O**_	**A**_**R**_	**P**_**A**_
1	Cala Lunga (Bonifacio, Corsica, France)	CCL	Tyrrhenian Sea	9\9\-	6	0.917	2.833	0.005	0.319	0.525	3.399	
2	Sutta-Rocca (Bonifacio, Corsica, France)	CSR	Tyrrhenian Sea	2\-\-	2	*-*	2.000	0.003				
3	Cala Reale (Asinara island, Sardinia, Italy)	SAR	West. Med. Sea	14\29\27	4	0.67	0.813	0.001	0.306	0.555	3.539	
4	Porto Pagliaccia (Asinara island, Sardinia, Italy)	SAP	West. Med. Sea	15\30\17	5	0.562	4.742	0.008	0.322	0.614	3.931	
5	Cala Barbarossa (Asinara island, Sardinia, Italy)	SAB	West. Med. Sea	14\30\12	5	0.758	0.989	0.002	0.337	0.600	3.521	
6	Molo Fornelli (Asinara island, Sardinia, Italy)	SAF	West. Med. Sea	11\16\-	8	0.927	8.09	0.014	0.313	0.522	3.517	
7	Saline (Stintino, Sardinia, Italy)	SSS	West. Med. Sea	15\30\-	6	0.79	1.295	0.002	0.524	0.657	4.244	
8	La Caletta (San Pietro island, Sardinia, Italy)	SPC	West. Med. Sea	10\23\18	6	0.911	7.088	0.012	0.356	0.576	3.747	
9	Cala Sapone (Sant’Antioco island, Sardinia, Italy)	SNS	West. Med. Sea	15\30\-	5	0.628	4.19	0.007	0.547	0.734	4.458	0.017(2)
10	Cala Lunga (Sant’Antioco island, Sardinia, Italy)	SNL	West. Med. Sea	1\-\14	1	*-*	0	0				
11	Spiaggia Grande (Sant’Antioco island, Sardinia, Italy)	SNG	West. Med. Sea	15\29\-	3	0.362	0.381	0.001	0.635	0.667	4.017	0.017(1)
12	Calasetta A (Sant’Antioco island, Sardinia, Italy)	SNA	West. Med. Sea	15\30\12	7	0.828	3.79	0.001	0.372	0.565	3.565	
13	Calasetta B (Sant’Antioco island, Sardinia, Italy)	SNB	West. Med. Sea	3\-\-	3	*-*	1.333	0.002				
14	Cussorgia (Sant’Antioco island, Sardinia, Italy)	SNC	West. Med. Sea	15\29\15	5	0.476	3.18	0.005	0.500	0.676	3.358	0.017(1)
15	Porticciolo (Cavoli island, Sardinia, Italy)	SCP	Tyrrhenian Sea	7\7\25	2	0.476	0.952	0.002	0.357	0.552	3.250	
16	Cala Ponente (Tavolara island, Sardinia, Italy)	STA	Tyrrhenian Sea	15\29\2	11	0.905	3.828	0.007	0.328	0.578	3.743	
17	Porto Massimo (Maddalena island, Sardinia, Italy)	SMM	Tyrrhenian Sea	13\15\23	7	0.833	4.564	0.008	0.330	0.578	3.409	0.167(1)
18	Lo Spalmatore (Maddalena island, Sardinia, Italy)	SMS	Tyrrhenian Sea	11\29\20	6	0.8	1.781	0.003	0.328	0.578	4.096	
19	Baia Trinità (Maddalena island, Sardinia, Italy)	SMT	Tyrrhenian Sea	15\30\21	5	0.771	1.657	0.003	0.449	0.607	4.037	
20	Porto (Maddalena island, Sardinia, Italy)	SMP	Tyrrhenian Sea	-\-\19								
21	Cala Portese (Caprera island, Sardinia, Italy)	SCA	Tyrrhenian Sea	-\-\18								
22	Telamone (Grosseto, Tuscany, Italy)	TTA	Tyrrhenian Sea	15\30\-	12	0.971	6.314	0.011	0.326	0.537	3.763	
23	S. Agostino (Civitavecchia, Latium, Italy)	LFM	Tyrrhenian Sea	6\-\-	5	0.933	5.133	0.009				
24	La Frasca (Civitavecchia, Latium, Italy)	FRA	Tyrrhenian Sea	14\30\105	9	0.835	8	0.014	0.371	0.504	3.676	
25	Dahlet Qorrot (Gozo island, Maltese Archipelago)	GOZ	East. Med. Sea	14\29\-	6	0.604	1.714	0.003	0.408	0.681	3.719	
26	Marina di Pulsano (Taranto, Apulia, Italy)	PMP	Ionian Sea	15\30\-	6	0.714	8.752	0.015	0.471	0.668	4.666	0.083(1)
27	Villaggio Nettuno (Lecce, Apulia, Italy)	PSF	Adriatic Sea	15\30\-	8	0.79	9.571	0.017	0.212	0.536	4.062	
28	San Cataldo (Lecce, Apulia, Italy)	PSC	Adriatic Sea	1\-\-	1	*-*	0	0				
29	Analipsi (Astipalea island, Greece)	GRE	Aegean Sea	-\-\15								

Number of sampled populations (Pop); sampling sites with corresponding codes; Mediterranean sub-basins; number of individuals analyzed (N) with mtDNA (COI), microsatellites (msat) and allozymes (alz); number of haplotypes (Hapl), and genetic indices for mtDNA (H = Gene diversity; *π* = Mean number of pairwise differences between pairs of Haplotypes; *π*_*n*_ = nucleotide diversity) and for microsatellites (H_E_ = Expected heterozygosity; H_O_ = Observed heterozygosity; A_R_ = Allelic richness; P_A_ = Private alleles: frequencies followed by the number in brackets). West. Med. Sea = Western Mediterranean Sea; East. Med. Sea = Eastern Mediterranean Sea.

The aim of this study is thus to determine the genetic structure of the beachflea *O. montagui* in the central portion of the Mediterranean Sea. The species is tightly linked to stranded heaps of the seagrass *P. oceanica* and hence passive dispersal via rafting is a plausible hypothesis. Here we specifically ask whether such passive dispersal in *O. montagui* is a strong enough factor to promote genetic homogeneity over long distances. *P. oceanica* wrack may provide food and shelter; given the wide distribution of this seagrass in the Mediterranean Sea we suspect events of transports to be quite frequent. On the other hand, being strictly associated to a potential vector of dispersal does not necessarily imply that gene flow is realized. We indeed found a remarkably deep geographic partition in the population genetic structuring of the talitrid *M. remyi*, a species strictly associated to stranded rotting logs that should theoretically aid passive dispersal [18]. Here we used multiple genetic data sets (mtDNA, microsatellites and allozymes), which allowed testing the following two alternative scenarios (marine and terrestrial, respectively). Under the marine scenario if passive dispersal at sea were predominant, we would expect patterns of genetic structuring to be relatively shallow within basins and largely congruent with those of marine Mediterranean taxa with planktonic stages in the life cycle. Multiple authors recurrently placed a major breakpoint to gene flow in marine species at the Strait of Sicily (STS): this separates the Western from the Eastern Mediterranean basin (the so-called STS boundary; [24-27]). We also expect sea surface currents - the main carriers of floating wracks - to shape patterns of relationships among populations. Alternatively, if the bound to terrestrial environment prevailed over dispersal at sea, we would expect more pronounced genetic divergence also at the within-basin level with evidence of genetic regionalism. Support for both scenarios was evaluated with Approximate Bayesian Computation [28]. Finally, the study represents an opportunity to compare three marker systems characterized by different evolutionary properties regarding inheritance (maternal for mtDNA vs. bi-parental for microsatellites and allozymes) and pace of molecular evolution (from relatively slow for the protein-coding allozymic loci to very fast for the non-coding microsatellites).

## Materials & methods

The study complies with the current laws of Italy and Germany regarding proper treatment of animals for research and was approved by the Ethical Committee of University of Rome "Sapienza", Italy.

### Sampling

*O. montagui* specimens were collected by hand or using an aspirator on *P. oceanica* decaying leaves. A total of 26 populations were sampled for this study on rocky and sandy beaches along shores of the following Mediterranean basins: Tyrrhenian Sea (10 populations), Western Mediterranean basin (12 populations), Eastern Mediterranean basin (one population), Ionian Sea (one population), Adriatic Sea (two populations). All 26 populations were analyzed at the mtDNA level and 21 (out of 26) were genotyped for microsatellites.

Additionally, we re-analyzed the dataset based on 21 allozymic loci of De Matthaeis et al. [8]. That study included 16 populations and 363 individuals. One population was from the Aegean Sea (an area that we could not cover with our new sampling) while 13 have been re-sampled for the present study. The different data sets combined hence include 29 populations: information about populations, sampling localities and markers used is listed in Table 1; all populations analyzed with mtDNA and microsatellites were collected anew for the study while De Matthaeis et al. [8] was the only source of the allozymic data. Figure 1 shows the geographic location of sampling sites.

### Genetic analyses

Including the 153 samples analyzed in Pavesi et al. [23], we extracted total genomic DNA from 544 individuals using the “Qiagen DNeasy Blood & Tissue Kit” (Hilden, Germany) according to the manufacturer’s protocol.

### Mitochondrial DNA

Folmer et al.’s [29] universal primers were used to amplify via Polymerase Chain Reaction (PCR) a 571 base pair long fragment of the mtDNA coding for the COI gene in 1 to 15 individuals per population for a total of 295 individuals (details are given in Table 1). COI double-stranded amplifications, purification of PCR products and sequencing reactions were carried out as described in [18,23,30]. Editing and alignment of sequences were performed using Sequencher 4.1 (Gene Codes, Ann Arbor, MI, USA) and checked by eye. We used Paup* v.4.0b10 [31] to calculate the number of parsimony-informative characters. We used Arlequin v.3.11 [32] to calculate genetic diversity (H), nucleotide diversity (*π*_*n*_) and the mean number of pairwise differences between all pairs of haplotypes (*π*). The same software was used to assess the population genetic structure by hierarchical analysis of molecular variance (AMOVA; [33]). Populations were combined according to their geographic origin and to the results of phylogeographic analyses (see Results). AMOVA was initially run on populations combined in three groups. These pooled all Tyrrhenian samples (populations 1–2 and 15–24 in Table 1), North Western Sardinian + Ionian and Adriatic samples (populations 3–7 and 26–28) and South Western Sardinian + Gozo Is. populations (8–14 and 25). An additional AMOVA was run on five groups of populations. These included Corsican + South Eastern Sardinian + Tyrrhenian peninsular sites (populations 1–2; 15 and 23–24), North Western Sardinian localities + Gozo Is. (populations 3–7 and 25), South Western Sardinian localities (populations 8–14), North Eastern Sardinian localities (populations 16–21) and Adriatic + Ionian samples (populations 26–28). We also calculated pairwise *F*_ST_ values on all pairs of populations as a measure of their differentiation. Statistical significance of these values was assessed by 1,000 permutations after sequential Bonferroni correction for multiple tests [34]. We also performed a Mantel Test [35] with Arlequin, to test for the presence of Isolation-by-distance (IBD) in the dataset; variables were pairwise *F*_ST_ values and corresponding geographic coordinates distances Degrees-Minutes-Seconds (DMS). Geographic distances were measured as the shortest marine distances between each pair of populations, according to the circulation of surface currents [36,37]. We used the spatial analysis of molecular variance (SAMOVA v.1.0) to detect genetic barriers among groups of populations that were geographically homogeneous and maximally differentiated from each other [38]. Arlequin was also used to assess the fit of demographic [39] and geographic [40] expansion models to *O. montagui* genetic variation (populations grouped according to the SAMOVA results). Mismatch distributions were used to fit the models of sudden demographic and geographic expansion and to estimate the τ parameter. In both models τ is a mutation-scaled measure of time since expansion (demographic or geographic) and can be used to estimate time in generations (T) since expansion, using T = τ/2 μ, where μ is the crustacean COI mutation rate per site per year of Knowlton et al. [41] already adopted in Pavesi et al. [23]. Goodness of fit was assessed by the sum of square deviations (SSD) between the observed and the expected mismatch, and its significance was determined by a parametric bootstrap with 10,000 replicates. Demographic equilibrium was also tested on each SAMOVA group by Tajima’s *D*[42] and Fu’s *F*_s_[43] statistics in Arlequin. These parameters have been shown to be sensitive to signals of population expansion; their statistical significance was tested by simulating random samples (10,000 replicates). *P*-values were obtained as the proportion of simulated values smaller than or equal to the observed values (α = 0.05).

In order to estimate genealogical relationships among all haplotypes, we used Tcs v.1.13 [44] to carry out a statistical parsimony analysis with a 95% connection limit [45]. To better visualize the haplotype network layout, we used HapStar [46] combined with Inkscape, a free open-source Svg graphics editor [47]. HapStar directly combines the network connection output data generated from Arlequin with a force-directed algorithm to automatically lay out the network for easy visualization.

### Microsatellites

We genotyped all 544 individuals (7 to 30 per population) at eight microsatellite loci (msats, Table 1). PCR amplifications were performed as described in [22,23]. Fragment sizes were determined on an ABI 3100 automatic sequencer using the GeneMapper v.3.5 software and an internal size standard (GS500LIZ from Applied Biosystems). We used Microchecker v.2.2.3 [48] to identify genotyping errors (null alleles) and the scoring of stutter peaks in all microsatellites. Null alleles can cause deviations from Hardy–Weinberg proportions. In order to test for the impact of null alleles on our data set, we calculated global *F*_ST_ values with FreeNA software. These values were computed, as described in Chapuis and Estoup [49], with 10,000 bootstrap iterations, alternatively using and not using the Excluding Null Alleles (ENA) method. We used Arlequin v.3.11 [32] to calculate expected (H_E_) and observed (H_O_) heterozygosity, deviations from Hardy–Weinberg equilibrium and tests of linkage disequilibrium among loci. We corrected *P*-values using the sequential Bonferroni correction for multiple comparisons [34]. We used Fstat v.2.9.3 [50,51] to calculate allelic richness (A_R_) for each population, and GenAlex v.6.3 [52] to determine the number of alleles per locus, the number of private alleles (P_A_) and their relative frequencies in each population. With Arlequin v.3.11 we calculated pairwise *F*_ST_ values and performed two AMOVA analyses (on three and five groups, respectively) and Mantel test as described for the mtDNA dataset in the previous paragraph. For the AMOVA we grouped populations based on the results of Structure v.2.3.2 [53] (three and five groups; see below).

We run Structure to evaluate genetic subdivision and to assign individuals to the most probable number of clusters (K). The software uses a Monte Carlo Markov Chain (MCMC) Bayesian clustering model that assumes that different loci are at Hardy-Weinberg and linkage equilibrium with one another within populations. We performed 10 independent runs for each value of K (K = 1–21) assuming an admixture model and using 100,000 replicates of the MCMC after a burn-in of 10,000 iterations. The number of clusters was calculated from the ΔK estimates using Evanno et al.’s method [54]. Assignments of individuals to the inferred clusters were estimated according to the highest Q-values (probability of membership).

We compared the results obtained from Structure with those from Tess v.2.3 [55]. The software is based on a hierarchical mixture model whose neighborhood system is obtained from the Voronoi tessellation [55]. We performed 10 runs (K = 2–21) with an admixture model with a total of 50,000 sweeps with a burn-in of 10,000. The number of clusters was calculated plotting the Deviance Information Criterion (DIC) against values of K and choosing the value of K that corresponded to a plateau of the curve [56].

### Allozymes

We re-analyzed the dataset of De Matthaeis et al. [8] consisting of 21 allozymic loci, 16 populations and 363 individual (2 to 105 per population; see Table 1). We calculated pairwise *F*_ST_ values, AMOVA and IBD using Arlequin. We performed the AMOVA on three groups combining populations according to their geographical position and patterns of relationships as detailed in De Matthaeis et al. [8] (Tyrrhenian + Aegean Sea; North Western Sardinia; South Western Sardinia). We analyzed the genetic structure of populations by running Structure (K = 1–16) and Tess (K = 2–16) with the settings described in the microsatellite section.

### Approximate Bayesian computation

We used Approximate Bayesian Computation in DIYABC 1.0.4.39 [57] to evaluate the two competing scenarios (marine vs. terrestrial) detailed in the Introduction. To reduce computation time, we selected nine populations (SCP, SNC, STA, SAR, CCL, FRA, GOZ, PMP, PSF) as representative of each sampled area. We chose populations with complete data sets for both mtDNA and microsatellites and with the lowest divergence from the other populations from the same area. The marine scenario was designed following the subdivision of the Mediterranean Sea in biogeographic sectors proposed by Bianchi & Morri [58]; these would group the West Mediterranean basin and the Tyrrhenian Sea together (hence SCP, SNC, STA, SAR, CCL, FRA were forced to merge into a common ancestor), the Maltese Archipelago (GOZ) would be a transition area between the former sector and the Ionian one (PMP) while the South Adriatic Sea (PSF) would form a separated biogeographic area. The terrestrial scenario was designed on the basis of the geographic origin of populations. We considered the following areas; North-Eastern (STA), North-Western (SAR), South-Western (SNC), South-Eastern Sardinia (SCP), Tyrrhenian Sea (CCL, FRA; one insular and one peninsular population), Maltese Archipelago (GOZ), Ionian (PMP) and Adriatic (PSF) Sea. Three data sets were considered in the analysis (mtDNA, microsatellites, both markers combined); for each scenario we simulated 2 × 10^6^ data sets. For mtDNA we assumed the default settings in DIYABC for mitochondrial markers; for microsatellites the loci were assumed to follow a generalized stepwise mutation model. We calculated the posterior probabilities of the two competing scenarios for each data set by *i*) evaluating the relative proportions of each scenario found in the selected closest data sets (Direct estimate; 500 simulated data sets) and through *ii*) a polychotomous logistic regression on the 1% of the simulated data sets closest to the observed data sets (default settings in DIYABC). Before estimating the posterior probabilities of scenarios, we checked whether the combination of scenarios and prior distributions of their parameters have produced data sets that are similar enough to the observed ones by performing a Principal Component Analysis (PCA) of the first 100,000 simulated data sets to evaluate the position of the observed data compared to the simulated.

## Results

### Mitochondrial DNA

The 295 COI sequences identified a total of 82 unique haplotypes (25 from Pavesi et al. [23] plus 57 newly found in this study) and 71 variable and parsimony-informative sites (GenBank Accession N.: JQ390313-JQ390337, KC568143-KC568199).

Values of H, *π*_*n*_ and *π* are listed in Table 1. H ranged between 0.362 (SNG) and 0.971 (TTA); *π* ranged between 0.381 (SNG) and 9.571 (PSF) whereas *π*_*n*_ ranged between 0.001 (SNA, SNG, SAR) and 0.017 (PSF). The AMOVA detected significant population structure when we tested for three and five groups (Table 2): in both cases the major source of variation was due to differences within populations. A small percentage of the detected variation was apportioned among groups; this increased when five groups were considered. Pairwise *F*_ST_ values are shown in Table 3: values ranged between 0.049 (SAF-TTA) and 0.596 (SSS-SNC). Most comparisons were significant after the Bonferroni correction with some exceptions. In particular, CSR (from Corsica Is.) diverged significantly from only two Sardinian populations (SSS and SNC) whereas the very close CCL (Corsica Is.) was significantly differentiated from it (and many others) but not from the far away PSC (Adriatic Sea). Significant was also the comparison between PMP and PSF (Ionian and Adriatic Sea, respectively). A Mantel test performed on all populations revealed no statistically significant relationship between genetic and geographic distances (R = 0.000005; *P* = 0.426).

**Table 2:** Analyses of molecular variance (AMOVA) performed on COI, microsatellites and allozyme data for K = 3 and K = 5 groups

	**COI**		**Microsatellites**		**Allozymes**	
**Source of variation**	**% of variation**	**Fixation index**	**% of variation**	**Fixation index**	**% of variation**	**Fixation index**
K = 3						
Among groups	1.27	0.012*	5.71	0.057***	3.73	0.034*
Among populations within groups	28.08	0.284***	11.28	0.119***	9.27	0.096***
Within populations	70.65	0.293***	83.01	0.169***	87.3	0.127***
K = 5						
Among groups	9.35	0.093**	7.6	0.076***		
Among populations within groups	21.04	0.232***	9.25	0.100***		
Within populations	69.61	0.303***	83.15	0.168***		

* *P* < 0.05; ** *P* < 0.01; *** *P* < 0.001.

**Table 3:** **Pairwise *F***_**ST **_**values for all comparisons among populations with mtDNA (upper diagonal), microsatellites (lower diagonal)**

	**CCL**	**CSR**	**SAR**	**SAP**	**SAB**	**SAF**	**SSS**	**SPC**	**SNS**	**SNL**	**SNG**	**SNA**	**SNB**	**SNC**	**SCP**	**STA**	**SMM**	**SMS**	**SMT**	**TTA**	**LFM**	**FRA**	**GOZ**	**PMP**	**PSF**	**PSC**
CCL	-	**0.061**	**0.217**	**0.280**	**0.168**	**0.078**	**0.289**	**0.152**	**0.086**	**0.242**	0.083	0.053	**0.330**	**0.399**	**0.090**	**0.127**	**0.144**	**0.162**	**0.131**	**0.054**	**0.076**	**0.127**	**0.254**	**0.194**	**0.152**	0.083
CSR	-	-	0.257	0.351	0.185	0.053	**0.401**	0.160	0.065	0.294	0.000	0.000	0.428	**0.536**	0.071	0.125	0.150	0.175	0.130	0.021	0.047	0.124	0.313	0.222	0.160	0.000
SAR	**0.242**	-	-	**0.385**	**0.286**	**0.207**	**0.408**	**0.269**	**0.217**	**0.351**	0.330	0.228	**0.429**	**0.487**	**0.211**	**0.249**	**0.268**	**0.278**	**0.250**	**0.178**	**0.221**	**0.247**	**0.363**	**0.307**	**0.269**	0.330
SAP	**0.145**	-	**0.073**	-	**0.341**	**0.267**	**0.471**	**0.324**	**0.278**	**0.405**	0.438	**0.313**	**0.481**	**0.538**	**0.267**	**0.307**	**0.328**	**0.333**	**0.305**	**0.233**	**0.292**	**0.303**	**0.417**	**0.362**	**0.324**	0.438
SAB	**0.161**	-	**0.057**	**0.040**	-	**0.160**	**0.357**	**0.225**	**0.170**	**0.308**	0.242	0.164	**0.385**	**0.444**	**0.168**	**0.205**	**0.222**	**0.235**	**0.206**	**0.134**	**0.168**	**0.203**	**0.319**	**0.264**	**0.225**	0.242
SAF	**0.231**	-	**0.177**	**0.126**	**0.162**	-	**0.274**	**0.144**	**0.081**	**0.231**	0.073	0.047	**0.314**	**0.378**	**0.084**	**0.121**	**0.136**	**0.154**	**0.124**	**0.050**	0.070	**0.120**	**0.242**	**0.185**	**0.144**	0.073
SSS	**0.166**	-	**0.091**	**0.063**	**0.066**	**0.153**	-	**0.337**	**0.287**	**0.431**	0.524	**0.344**	**0.524**	**0.596**	**0.274**	**0.318**	**0.342**	**0.348**	**0.316**	**0.238**	**0.306**	**0.314**	**0.446**	**0.381**	**0.337**	0.524
SPC	**0.149**	-	**0.151**	**0.114**	**0.117**	**0.211**	**0.134**	-	**0.153**	**0.290**	0.210	0.141	**0.367**	**0.424**	**0.152**	**0.189**	**0.205**	**0.219**	**0.190**	**0.119**	**0.150**	**0.187**	**0.301**	**0.248**	**0.210**	0.210
SNS	**0.143**	-	**0.112**	**0.096**	**0.094**	**0.159**	**0.119**	**0.043**	-	**0.241**	0.089	0.057	**0.327**	**0.393**	**0.092**	**0.129**	**0.145**	**0.163**	**0.133**	**0.057**	**0.079**	**0.129**	**0.253**	**0.194**	**0.153**	0.089
SNL	-	-	-	-	-	-	-	-	-	-	0.371	**0.261**	**0.448**	**0.505**	**0.233**	**0.272**	**0.291**	**0.300**	**0.271**	**0.200**	**0.249**	**0.270**	**0.383**	**0.329**	**0.290**	0.371
SNG	**0.143**	-	**0.125**	**0.107**	**0.127**	**0.159**	**0.137**	**0.043**	**0.074**	-	-	0.000	0.524	0.638	0.095	0.167	0.200	0.229	0.171	0.029	0.067	0.165	0.396	0.286	0.210	1.000
SNA	**0.188**	-	**0.122**	**0.093**	**0.113**	**0.148**	**0.131**	**0.060**	**0.055**	-	**0.020**	-	0.383	**0.481**	0.063	0.110	0.132	0.155	0.115	0.018	0.040	0.110	0.278	**0.196**	0.141	0.000
SNB	-	-	-	-	-	-	-	-	-	-	-	-	-	**0.581**	**0.310**	**0.351**	**0.375**	**0.376**	**0.348**	**0.276**	**0.349**	**0.347**	**0.461**	**0.405**	**0.367**	0.524
SNC	**0.218**	-	**0.181**	**0.165**	**0.187**	**0.181**	**0.195**	**0.151**	**0.132**	-	**0.018**	**0.049**	-	-	**0.367**	**0.411**	**0.438**	**0.433**	**0.405**	**0.333**	**0.427**	**0.406**	**0.519**	**0.462**	**0.424**	0.638
SCP	0.070	-	**0.206**	**0.112**	**0.153**	**0.186**	**0.156**	**0.117**	**0.100**	-	**0.100**	**0.127**	.	**0.167**	-	**0.130**	**0.145**	**0.162**	**0.133**	**0.062**	**0.083**	**0.130**	**0.244**	**0.190**	**0.152**	0.095
STA	**0.118**	-	**0.197**	**0.126**	**0.169**	**0.159**	**0.128**	**0.175**	**0.122**	-	**0.174**	**0.164**	-	**0.216**	**0.090**	-	**0.183**	**0.198**	**0.169**	**0.096**	**0.123**	**0.166**	**0.283**	**0.228**	**0.189**	0.167
SMM	**0.196**	-	**0.210**	**0.125**	**0.166**	**0.155**	**0.165**	**0.131**	**0.129**	-	**0.129**	**0.165**	-	**0.227**	**0.163**	**0.139**	-	**0.215**	**0.185**	**0.111**	**0.141**	**0.182**	**0.303**	**0.245**	**0.205**	0.200
SMS	**0.152**	-	**0.160**	**0.115**	**0.160**	**0.145**	**0.118**	**0.139**	**0.095**	-	**0.157**	**0.148**	-	**0.197**	**0.152**	**0.070**	**0.097**	-	**0.200**	**0.129**	**0.161**	**0.197**	**0.311**	**0.257**	**0.219**	0.229
SMT	**0.098**	-	**0.185**	**0.091**	**0.108**	**0.170**	**0.139**	**0.079**	**0.092**	-	**0.140**	**0.131**	-	**0.196**	**0.132**	**0.134**	**0.074**	**0.096**	-	**0.100**	**0.127**	**0.168**	**0.282**	**0.229**	**0.190**	0.171
TTA	**0.064**	-	**0.210**	**0.139**	**0.186**	**0.164**	**0.181**	**0.186**	**0.145**	-	**0.145**	**0.164**	-	**0.155**	0.051	**0.095**	**0.188**	**0.142**	**0.152**	-	0.045	**0.096**	**0.210**	**0.157**	**0.119**	0.029
LFM	-	-	-	-	-	-	-	-	-	-	-	-	-	-	-	-	-	-	-	-	-	**0.123**	**0.263**	**0.196**	**0.150**	0.067
FRA	**0.255**	-	**0.282**	**0.279**	**0.239**	**0.323**	**0.273**	**0.275**	**0.215**	-	**0.285**	**0.304**	-	**0.336**	**0.245**	**0.237**	**0.219**	**0.204**	**0.220**	**0.232**	-	-	**0.280**	**0.226**	**0.187**	0.165
GOZ	**0.118**	-	**0.117**	**0.061**	**0.101**	**0.036**	**0.116**	**0.143**	**0.095**	-	**0.095**	**0.091**	-	**0.114**	**0.087**	**0.100**	**0.132**	**0.111**	**0.109**	**0.076**		**0.254**	-	**0.340**	**0.301**	0.396
PMP	**0.207**	-	**0.112**	**0.119**	**0.119**	**0.173**	**0.112**	**0.163**	**0.113**	-	**0.175**	**0.146**	-	**0.230**	**0.170**	**0.137**	**0.192**	**0.149**	**0.170**	**0.208**	-	**0.278**	**0.043**	-	**0.248**	0.286
PSF	**0.293**	-	**0.141**	**0.175**	**0.160**	**0.221**	**0.183**	**0.222**	**0.158**	-	**0.210**	**0.185**	-	**0.258**	**0.248**	**0.207**	**0.253**	**0.222**	**0.245**	**0.261**	-	**0.340**	**0.178**	**0.138**	-	0.210
PSC	-	-	-	-	-	-	-	-	-	-	-	-	-	-	-	-	-	-	-	-	-	-	-	-	-	-

Refer to main text and Table 1 for details on population codes. Values highlighted in bold indicate statistical significance at the *P* < 0.05 level after Bonferroni correction.

SAMOVA suggested the existence of three groups (*F*_CT_ = 0.45; *P* < 0.05), finding two barriers isolating the Adriatic from the Ionian populations and then these two basins from the rest. The analysis of mismatch distributions supported demographic expansion in the Adriatic group (*P*_SSD_ = 0.283) only; geographic expansion was supported in all groups (*P*_SSD_ ≥ 0.184). Tajima’s *D* and Fu’s *F*_s_ were not significant for the Ionic (*D* = −0.792, *P*_*D*_ = 0.217; *F*_S_ = 3.854, *P*_*F*S_ = 0.95) and Adriatic (*D* = −1.468, *P*_*D*_ = 0.06; *F*_S_ = 0.932, *P*_*F*S_ = 0.706) groups while they were negative and highly significant for the remaining populations lumped in a single group (*D* = −2.157, *P*_*D*_ = 0.000; *F*_S_ = −25.189, *P*_*F*S_ = 0.000). In light of the relatively poor performance of mismatch distribution statistics in detecting population demographic expansion compared to *D* and *F*_S_ statistics [59], we tend to favor the latter two, which support demographic expansion in the Western Mediterranean and Tyrrhenian populations. In the Adriatic group the timing of demographic expansion (τ = 34.63; T = 1.731 million years) preceded the spatial expansion (τ = 7.52; T = 376,000 years). In the Ionic basin the geographic expansion dates back to 500,000 years ago (τ = 10). In the Western Mediterranean and Tyrrhenian Sea the two expansions were more recent and coeval (τ = 1.01; T = 50,500 years), justifying the star-like shape of the haplotype network (see below). The relatively small τ values suggest that expansions always started from small effective population sizes.

**Figure 2:**
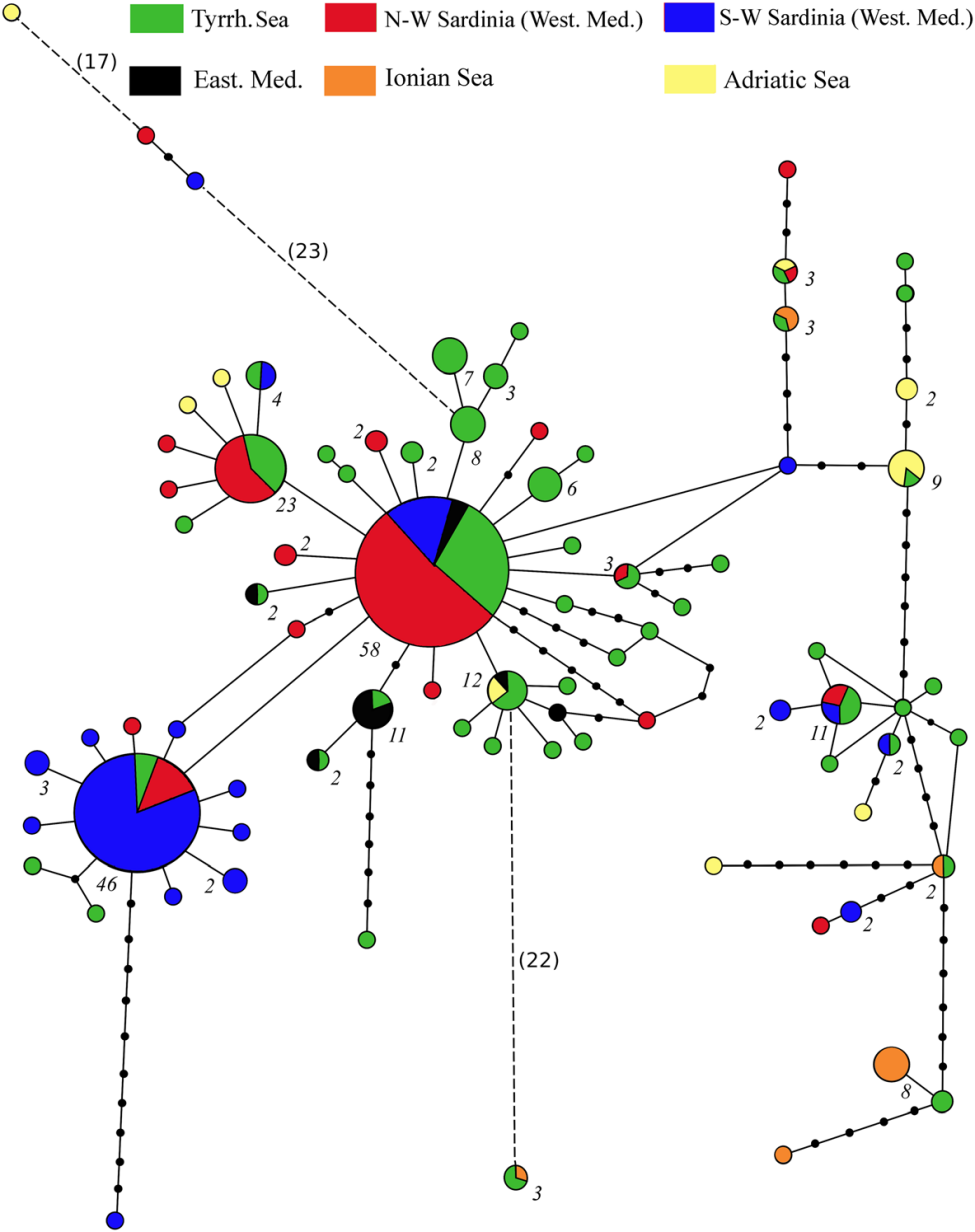
**Statistical parsimony haplotype network of COI (mtDNA) haplotypes.** The size of each circle is proportional to the haplotypes frequency; numbers beside circles refer to the number of individuals carrying that haplotype (N = 1 when no number is indicated). Black dots represent missing haplotypes; haplotypes are one mutational step away from one another if not indicated otherwise. Different colors identify the geographic origin: green – Tyrrhenian Sea; red – Northern-Sardinia (Western Mediterranean Sea); blue – Southern Sardinian (Western Mediterranean Sea); black – Eastern Mediterranean Sea; orange – Ionian Sea; yellow– Adriatic Sea.

The statistical parsimony network is shown in Figure 2. Altogether, 53 out of 82 haplotypes were singletons. The analysis yielded one network linking all haplotypes. Three main haplogroups could be recognized: two haplogroups were star-like shaped and most of the sampled Mediterranean basins were represented in there with the exception of the Ionian and Adriatic ones. These were instead loosely gathered in a third sub-group, together with some Tyrrhenian and Western Mediterranean haplotypes and many intervening missing haplotypes. In four circumstances rare haplotypes from different basins were very divergent from all others, being separated by a high number of substitutions (bracketed in Figure 2).

### Microsatellites

All microsatellite loci were polymorphic in the 21 studied populations with the number of alleles per locus ranging from 7 to 16. Number of individuals analyzed, H_E_ and H_O_, A_R_ and P_A_ are provided in Table 1. No scoring errors and no evidence of linkage disequilibrium were detected between any pairs of loci after Bonferroni correction. For some loci Microchecker indicated possible presence of null alleles; global *F*_ST_ values calculated alternatively including and excluding null alleles returned very similar values (Table 4) suggesting very little effect of null alleles on *F*_ST_ estimates.

**Table 4:** **Global *F***_**ST **_**values at eight microsatellite loci calculated using FreeNA software and employing 10000 bootstrap iterations**

**Locus**	**Global *F***_**ST **_**not using ENA**	**Global *F***_**ST **_**using ENA**
1	0.301606	0.280849
2	0.065796	0.080518
3	0.119156	0.118377
4	0.159405	0.141352
5	0.114114	0.101403
6	0.146024	0.125829
7	0.124407	0.118899
8	0.099773	0.082684

AMOVA indicated a significant genetic structure and most of the genetic variability, when populations were grouped either in three (83.01%) or five groups (83.15%), was apportioned within populations (Table 2). Population differentiation (pairwise *F*_ST_, Table 3) was significant for all pairwise comparisons except two comparisons (TTA-SCP and CCL-SCP). The Mantel test did not detect a pattern of IBD (R = 0.0001; *P* = 0.465).

**Figure 3:**
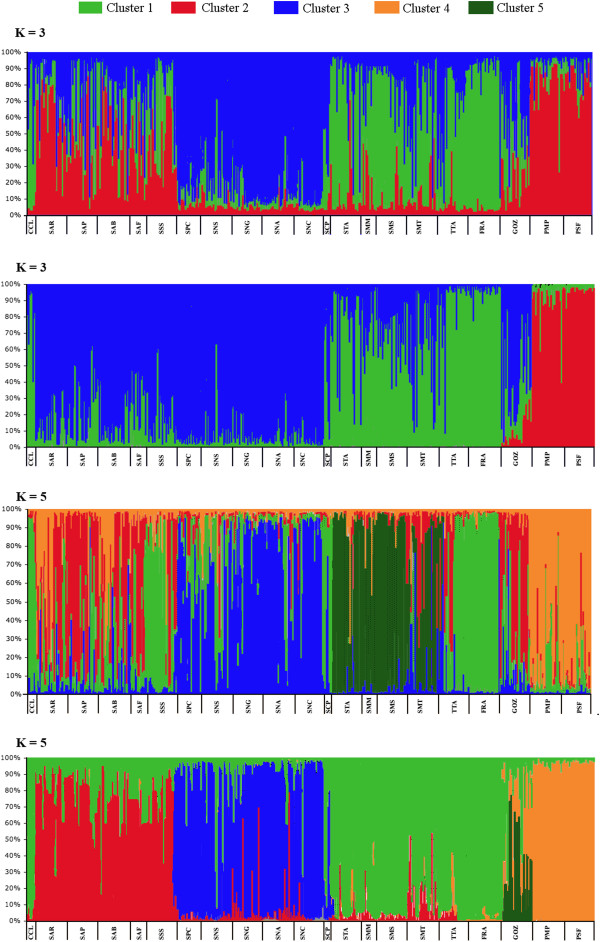
**Patterns of genetic divergence at the eight microsatellite loci as revealed by Structure (above) and Tess (below) for K = 3 and K = 5.** Each vertical line represents one individual. The length of each line reflects the percentage probability of each individual’s membership to each cluster. For K = 3 clusters are shown in green, blue and red. For K = 5 clusters are indicated in light green, red, blue, orange and dark green. Codes for populations are as in Table 1.

Results from Structure and Tess indicated respectively K = 3 (LnP(K) = −13309.7) and K = 5 (DIC = 18998.5) as the best fitting our data (Figure 3). We also analyzed results for K = 5 in Structure (LnP(K) = −13310.1) and K = 3 (DIC = 20242.1) in Tess for a better visualization of data and further comparison. For K = 3 in Structure, the three clusters corresponded to 1) Western Mediterranean basin (Northern Sardinian populations: SAR-SAP-SAB-SAF-SSS) plus Ionian and Adriatic populations (PMP and PSF); 2) Tyrrhenian basin (STA-SMM-SMS-SMT-TTA-FRA-CCL); 3) Western Mediterranean basin (Southern Sardinian populations: SPC-SNS-SNG-SNA-SNC) plus a single population from the Tyrrhenian basin (SCP). The Eastern Mediterranean population (GOZ; Maltese Archipelago) was shared between cluster 1 and 3. Tess with K = 3 also retrieved the Tyrrhenian cluster, but the Ionian and Adriatic populations clustered on their own while the third cluster grouped all populations from the Western Mediterranean basin plus GOZ (Eastern Mediterranean basin).

With K = 5 Tess identified the following five geographic clusters: 1) Northern Sardinian populations (Western Mediterranean basin); 2) Southern Sardinian populations (Western Mediterranean basin), 3) Tyrrhenian basin, 4) GOZ (Maltese Archipelago; Eastern Mediterranean basin), 5) Ionian plus Adriatic basins. When Structure was forced to use K = 5, the software also split the Western Mediterranean populations in two groups (corresponding to Northern and Southern Sardinian locations). Additional groups were formed by the Ionian and the Adriatic populations together and by two Tyrrhenian clusters. The Tyrrhenian groups corresponded to North Eastern Sardinian islets (populations STA, SMM, SMS and SMT) and Italian peninsular sampling sites (TTA, FRA and SCP).

Intriguingly, individuals carrying the four deeply divergent COI haplotypes (Figure 2) do not possess a similarly deviant microsatellite genotype. Membership of the Tyrrhenian/Ionian, S-W Sardinian, and Adriatic individuals to the respective clusters ranges between 0.84 and 0.98 (Structure and Tess). Tess assigned the N-W Sardinian individual to the Northern Sardinian group with a membership of 0.75 while Structure suggested for this individual a membership shared between cluster 1 and 2 (0.36, 0.43).

### Allozymes

We re-analyzed a dataset of 16 populations (13 of them in common with our data set; Table 1) genotyped at 21 allozyme loci by De Matthaeis et al. [8]. Pairwise *F*_ST_ values ranged between 0.03 (FRA-SNA) and 0.49 (STA-SAP) (Table 5). STA was significantly differentiated from all populations, whereas the Greek population (GRE) was significantly differentiated only from STA (Tyrrhenian basin). Northern and Southern Sardinian populations (Western Mediterranean basin) were consistently differentiated from one another with the exceptions of SPC (Southern Sardinia). Interestingly, significant differentiation emerged also at a scale as low as that of the Asinara Is., where SAR was found to be significantly divergent from both SAP and SAB. The AMOVA analysis revealed that 87.3% (*P* = 0.0001) of the genetic variance was apportioned within populations. A Mantel test revealed a lack of significant IBD when the Greek population (GRE) was excluded from the analysis (R = 0.0001; *P* = 0.006), whereas IBD was detected when GRE was included in it (R = 0.0001; *P* = 0.001).

**Table 5:** **Pairwise *F***_**ST **_**values for all comparisons among populations with allozymes**

	**SAR**	**SAP**	**SAB**	**SPC**	**SNL**	**SNA**	**SNC**	**SCP**	**STA**	**SMM**	**SMS**	**SMT**	**SMP**	**SCA**	**FRA**	**GRE**
SAR	-															
SAP	**0.055**	-														
SAB	**0.034**	0.022	-													
SPC	0.032	0.011	−0.007	-												
SNL	**0.066**	**0.102**	**0.112**	**0.076**	-											
SNA	**0.101**	**0.169**	**0.165**	**0.132**	−0.016	-										
SNC	**0.060**	**0.096**	**0.102**	**0.080**	−0.018	−0.006	-									
SCP	**0.068**	**0.109**	**0.096**	**0.062**	−0.015	−0.012	−0.007	-								
STA	**0.257**	**0.492**	**0.418**	**0.413**	**0.438**	**0.472**	**0.406**	**0.433**	-							
SMM	**0.054**	0.015	0.031	0.014	0.043	**0.084**	0.040	0.038	**0.429**	-						
SMS	**0.105**	**0.164**	**0.164**	**0.128**	−0.005	−0.015	0.008	−0.003	**0.449**	**0.083**	-					
SMT	**0.049**	0.027	0.022	0.000	**0.059**	**0.111**	**0.059**	**0.050**	**0.418**	0.003	**0.098**	-				
SMP	**0.062**	0.058	**0.101**	**0.062**	−0.003	**0.050**	0.017	0.035	**0.469**	0.043	0.055	**0.045**	-			
SCA	**0.056**	−0.008	**0.053**	0.025	0.051	**0.121**	0.063	**0.080**	**0.491**	0.026	**0.121**	0.028	0.002	-		
FRA	**0.122**	**0.191**	**0.173**	**0.155**	**0.054**	**0.033**	0.027	0.028	**0.388**	**0.111**	0.029	**0.130**	**0.131**	**0.180**	-	
GRE	0.012	0.152	0.063	0.023	0.093	0.194	0.071	0.104	**0.387**	0.034	0.154	−0.032	0.025	0.044	0.164	-

Refer to main text and Table 1 for details on population codes. Values highlighted in bold indicate statistical significance at the *P* < 0.05 level after Bonferroni correction.

**Figure 4:**
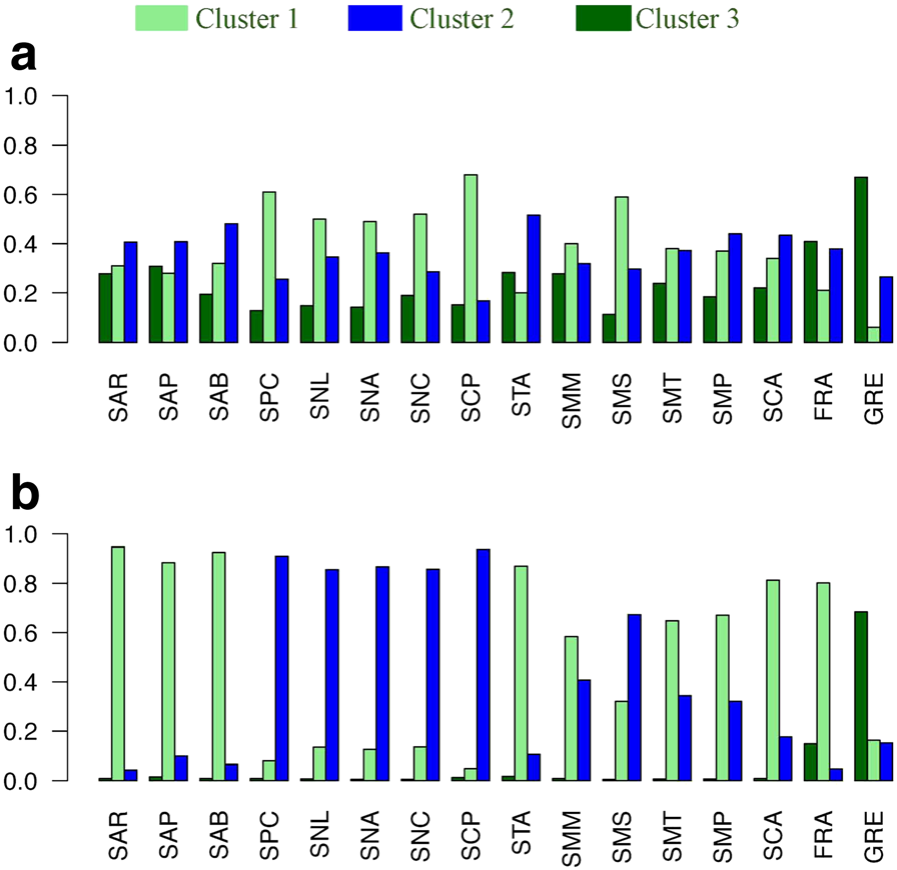
**Patterns of genetic divergence at twenty-one allozymic loci. a**,**b** Allozymes results for Structure (**a**) and Tess (**b**) (K = 3). Histograms show the average membership to one of the three clusters of each population (see Table 1 for details). Clusters are indicated by light green, blue and dark green and correspond to Tyrrhenian Sea and Northern Sardinia (Western Mediterranean Sea) (light green), Southern Sardinia (Western Mediterranean Sea) (blue) and Aegean Sea (dark green) (see text for details).

Both Structure and Tess analyses performed on this dataset revealed K = 3 ((LnP(K) = −1767.52 and DIC = 2887.23 respectively) as the best describing the species population structuring (Figure 4a, b). Nevertheless, membership of individuals to the inferred clusters was always very low in Structure and no real structuring was evident (Figure 4a). Results from Tess (Figure 4b) gave instead a clear subdivision in three clusters. A first one included populations located in the northern (respect to our samplings) part of the Tyrrhenian (populations FRA, SMP, SMT, SMM, SCA, STA) and Western Mediterranean Sea (populations SAP, SAB, SAR); a second cluster grouped populations from the southern part of the Tyrrhenian (SCP) and the Western Mediterranean Seas (SNC, SNL, SNA, SPC). A population from the Maddalena Is. (SMS, Tyrrhenian Sea) was included in the second cluster in spite of the northern position of the island respect to Sardinia (see Figure 1). Memberships of these populations to this cluster were low. The third cluster included GRE only (Aegean Sea). Finally, to further compare microsatellite and allozyme data we ran the latter in Structure and Tess enforcing K = 5 (results not shown graphically). In Structure no pattern emerged (membership Q < 0.4 in all cases except GRE); Tess couldn’t detect any additional cluster, as individuals were partitioned again among the three above-mentioned clusters.

### Approximate Bayesian Computations

**Figure 5:**
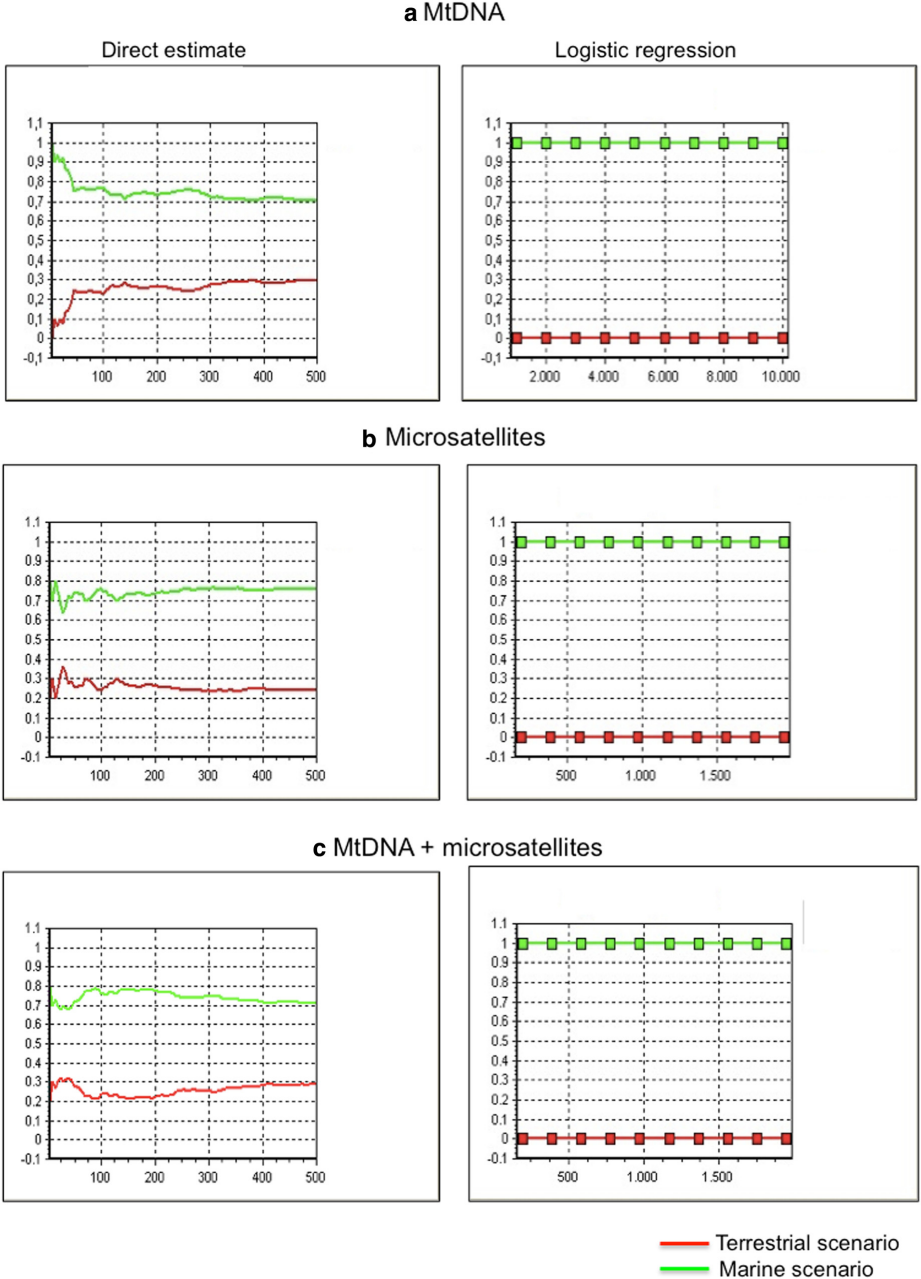
**Results of the Approximate Computation Analyses. a**,**b**,**c** ABC results for mtDNA (**a**), microsatellites (**b**) and both marker systems combined (**c**). On the left are shown the posterior probabilities based on direct estimates, on the right posterior probabilities are obtained through a logistic regression. In all circumstances the marine scenario is better supported than the terrestrial one.

The PCA analyses conducted to check whether the combination of scenarios and prior distributions of their parameters have produced data sets similar enough to the observed ones revealed in all circumstances that observed data were surrounded by many simulated data (not shown). Figure 5a, b, c shows the probabilities calculated with the two approaches (direct estimate and logistic regression) for both scenarios and for each marker system separately and together. Results are remarkably congruent across data sets and approaches; ABC consistently identified the marine scenario as largely supported over the terrestrial one.

## Discussion

In the last two decades we have been accumulating genetic data on Mediterranean talitrids with the aim to comparatively describe the genetic structure of different species at the scale of the whole Mediterranean Basin (see [60] and references therein for a review on the issue). So far, the present contribution is the largest one both in terms of individuals screened and number of markers used focused on a single talitrid species.

Present genetic data suggest that the beachflea *O. montagui* has a complex phylogeographic structure. By using ABC, we found that a scenario with marine forces assumed as predominant in shaping the species genetic structure is clearly better supported than a scenario of regionalism based on the geographic origin of populations (terrestrial scenario). The marine scenario is favored regardless the marker system analyzed (mtDNA and microsatellites; either separately or combined). It is worth noting that, in spite of this robust evidence, patterns of relationships yielded by mtDNA and microsatellites (and also allozymes but to a lesser extent) are not mirroring one another but major differences emerged that can be explained considering the intrinsically different evolutionary properties of these markers.

Populations east and west of the theoretical STS boundary are significantly differentiated at the mtDNA level (SAMOVA results) while microsatellites are ambivalent on the issue (compare the most likely groupings identified by Structure and Tess; three and five respectively). The population sampled on Gozo Is. (Maltese Archipelago; Eastern Mediterranean basin) shares both mtDNA haplotypes and microsatellite alleles with Western Mediterranean, Tyrrhenian, Ionian and Adriatic populations. The lack of isolation by distance in the mtDNA and both nuclear datasets excludes geographic distance as the main determinant of genetic divergence. On the other hand, the vast majority of pairwise *F*_ST_ values and the AMOVA results are statistically significant regardless of the marker considered. This evidence points to an overall scenario of complex genetic fragmentation that needs to be carefully evaluated to correctly resolve population structure in the species.

As pointed out by Thiel and Haye [61], population connectivity of rafters depends on a variety of factors (distances between habitats, sea current patterns, dispersal capability and colonization potential of organisms) and unraveling the relative importance of each one is a hard task that would require coupling genetic and field data. *O. montagui* is a small enough organism to hypothesize that it could cling, find shelter and feed on *P. oceanica* wrack while at sea for as long as the wrack would persist and/or strand in a new location. The reproductive biology of amphipods may enhance dispersal and recruitment; by incubating the embryos and developing juveniles in the brood pouch females provide extended parental care [62]. Migrating gravid females would favor persistence of rafters over generations. The four deeply divergent COI haplotypes that we found in our dataset (Figure 2) could indeed represent immigrants from geographical areas that we did not cover with our sampling. The mtDNA legacy of these alleged immigrants has persisted in five locations due to the mtDNA maternal inheritance while their ancestry has vanished at the microsatellite level due to the bi-parental inheritance of these markers. The sporadic occurrence of haplotypes profoundly divergent from an otherwise locally homogeneous set of lineages has been already reported in the talitrid *T. saltator*[17].

In spite of this convincing evidence, we suggest caution in interpreting the previous and following findings in light of our incomplete sampling coverage in the Eastern Mediterranean Sea and the small sample size for one Adriatic population.

### Divergence among and within basins: historical vs. contemporary processes

The SAMOVA analysis conducted on mtDNA data detected barriers to gene flow between Adriatic and Ionian populations and between these two and the remaining basins. Patterns of mtDNA relationships within each of the above groups are not similar. Adriatic and Ionian haplotypes are scattered in the network of Figure 2 and many intervening missing haplotypes need to be postulated to connect them. Conversely, the vast majority of haplotypes from the Tyrrhenian, Western and Eastern Mediterranean basins clusters around the two most frequent mtDNA variants and differs from them by one single mutational step. This indicates that haplotypes coalesce earlier into their last common ancestor in the Tyrrhenian, Western and Eastern Mediterranean basins than they do in the Adriatic and Ionian ones. A variety of reasons either intrinsic to the species ecology (different rates of population crashes) or basin-specific (differences in rates of formation of favorable conditions, i.e. heaps of stranded seagrasses, intermittent rafting routes, marine genetic barriers) could be responsible. Field studies aimed to gain a detailed understanding of the factors regulating the species’ niche are needed to properly understand the causes of these alternate mtDNA patterns.

Western Mediterranean/Tyrrhenian, Ionian and Adriatic mtDNA clusters (i.e. the three groups recognized by SAMOVA but see also ABC results) perfectly overlap with the corresponding biogeographic provinces identified within the Mediterranean Sea [58]; this evidence suggests historical factors to be responsible for the mtDNA pattern. Barriers to mtDNA gene flow can be traced back to the Pleistocene [23], when the sea level repeatedly dropped at each ice age peak and the different Mediterranean basins shrunk and became largely isolated from one another ([58] and references therein). The demographic and geographic expansions detected in our mtDNA data sets are indeed recurrently placed after major drops in sea level [63].

Microsatellites (and to a lesser extent allozymes) cluster individuals preferentially concordant to their geographic origin, while many mtDNA haplotypes from geographically far away locations are recurrently gathered together in the haplotype network and differ by one or few substitutions. The geographically close Ionian and Adriatic populations have quite similar microsatellite profiles (Figure 3) whereas divergence emerges between Northern and Southern Western Sardinian populations. Divergence was detected also among the Tyrrhenian, Western and Eastern Mediterranean basins. A closer look at the haplotype network of Figure 2 reveals that Northern and Southern Western Mediterranean populations have inverted frequencies of the two most common haplotypes, giving partial support (yet not detected by SAMOVA) to the microsatellite evidence. Altogether, patterns of relationships at the mtDNA level have relatively less geographical structure than those yielded by microsatellites. How could we then reconcile these discrepancies in light of the inherently different evolutionary properties of mtDNA and microsatellites [64]? The finer degree of microsatellite structuring would argue in favor of a female-biased gene flow. In our recent study on *O. montagui* based on a subset of six populations, we invoked male-biased dispersal to explain why the mtDNA fragmentation was not apparent at the microsatellite level [23]. In the current study, which relies on a better coverage of the species geographic distribution, the pattern is reversed. In light of this, we tend here to favor the female-biased gene flow hypothesis. This would also be in agreement with field studies showing that populations of Mediterranean talitrids, including *O. montagui*, generally have a strongly female-biased sex ratio ([65] and references therein).

*O. montagui* is able to disperse actively only over short distances; individuals could nonetheless be dragged away by waves within floating banks of *P. oceanica* and – theoretically –carried over ample ranges. The pattern of genetic structuring yielded by microsatellites is, in our opinion, reflecting these present-day processes and is influenced by the surface circulation of water masses, which are the principal carriers of floating material at sea. Genetic connectivity mirrors quite remarkably patterns of Mediterranean surface currents. Water masses flow in a south–north direction along the Tyrrhenian coast of the Italian peninsula and bifurcate along the Tuscan coasts into two branches (see Figure 1 modified from [37]). The first branch flows northward along the eastern coast of Corsica while a second one moves southward following the Sardinian eastern coasts. This pattern would explain why microsatellites consistently cluster Tyrrhenian populations together. Along the Sardinian western coasts water masses flow in a northward direction and a major gyre exists in the area, which is likely the cause of the microsatellite divergence found between South- and North-Western Sardinian populations (Western Mediterranean basin). The currents flowing in and out of the Adriatic Sea would promote gene flow between the Adriatic and Ionian populations. Finally, the highly admixed genetic profile of the sole Eastern Mediterranean population included in the study (GOZ) might be due to the position of the Maltese Archipelago at the crossroads between the main current flowing from the Atlantic Ocean to the Aegean Sea and that descending along the Ionian and South-eastern Sicilian coasts. The former would carry immigrants of Western Mediterranean and Tyrrhenian origin while the latter would be the source of Ionian and Adriatic alleles.

### Comparisons to other Mediterranean species

We could not detect a decrease in genetic variability from the Western to the Eastern Mediterranean Sea, as previously shown by Viñas et al. [66] in swordfish. Multi-taxa evidence supports a marked decrease in variability between the Mediterranean and the Black Sea but not necessarily in a west–east direction within the Mediterranean Sea [67].

When we compare our data within those available on a similar geographic scale for other Mediterranean species an interesting result emerges. Several marine species are genetically more homogeneous in the Western Mediterranean basin than they are in the Eastern Mediterranean, Ionic or Adriatic Sea. This was recurrently observed in fishes (i.e. gobids; with Adriatic, Aegean and Tunisian groups, [25]; anchovies, with a clear distinction between Adriatic, Aegean and the rest of Mediterranean Sea, [68]). *O. montagui* partially follows this scheme; the uniqueness of the Adriatic and Ionic populations is evident at the mtDNA level only. Likewise, Western and Tyrrhenian basins are shallowly fragmented at the mtDNA level while microsatellites revealed within-groups patchiness. Interestingly enough, allozymes detected a fixed alternative allele between the Aegean population (GRE) and all the others analyzed in [8]. Unfortunately, since we did not have access to any samples from the Aegean Sea for mtDNA or microsatellites, we cannot be conclusive on the issue.

Previous studies on Mediterranean talitrid amphipods coarsely found that species with a profound population genetic structure are generally confined to sandy beaches where they burrow into sand (i.e. *T. saltator*, *D. deshayesii*) or dig into rotting logs (*M. remyi*) [8,15,18,60]. Various species of *Orchestia*, including *O. montagui*, associated with heaps of decaying wrack were thought to be genetically homogeneous, even across vast geographical ranges [8-15]. The spatial scale at which genetic divergence would become apparent was found to vary considerably in talitrids, from few kilometers with no sharing of mtDNA haplotypes in *M. remyi*[18] to hundreds of kilometers in *O. montagui* ([8]; allozyme data). In *T. saltator*, allozymes clearly distinguished reciprocally isolated Tyrrhenian, Adriatic and Aegean groups [8].

The intrinsic active mobility of organisms is only one determinant of the amount of the realized dispersal [69]. Active dispersal is always limited in talitrids, independently from the single species’ ecology. Hence, differences in their degree of genetic structuring must have arisen because of differences in the amount of passive dispersal the various species are able to achieve. *M. remyi* lives in strong association with rotting logs stranded on sandy beaches; *T. saltator* burrows deeply into the sand to escape dehydration. In both circumstances, the chances for individuals to be dragged away by waves are far lower than those of *O. montagui*, which colonizes a temporally ephemeral habitat close to the water line. This would explain why our focus species was thought to be genetically poorly structured [8-15]. On the other hand, we believe that the conclusions to which previous studies on Mediterranean talitrids have arrived should be critically evaluated, especially for those species identified as able to sustain high levels of gene flow at the scale of the whole Mediterranean Sea. We have here shown that *O. montagui* can no longer be considered a species with a shallow genetic structure, as De Matthaeis et al. [8-15] concluded on allozyme basis. We have demonstrated that long distance dispersal through surface currents might happen in this species but these episodes are not frequent enough to ensure high levels of gene flow that would counteract the onset of appreciable local differences. A possible limiting factor could be identified in the relatively short persistence in time of the vector of passive dispersal (the *P. oceanica* wrack) at sea surface due to its negative buoyancy [61].

By comparing patterns yielded by microsatellites and allozymes for the same set of populations, we obtained an identical number of clusters (3) but membership of individuals was consistently higher in the microsatellite analyses, pointing – as expected – to a much better resolving power of these markers. The molecular footprint of high gene flow under low-resolution molecular assays that *O. montagui* was believed to bear has in fact converted to an appearance of intermediate gene flow under more stringent assays.

## Conclusions

We were able to unveil the genetic structuring of *O. montagui* within the Mediterranean Sea at a fine geographical scale. By combining molecular markers with different evolutionary properties we distinguished historical processes from present-day forces. Pleistocene climatic changes caused the isolation and divergence of mtDNA lineages. Current dynamics – namely surface marine currents jointly with rafting via seagrass wrack – produced new and different patterns of structuring, which were revealed by microsatellites. ABC gave strong support to a scenario of genetic fragmentation molded predominantly by marine forces.

The lack of a statistically significant pattern of IBD at both classes of markers under a scenario of genetic fragmentation (see *F*_ST_ and AMOVA results) suggests that the species has not yet approached equilibrium between gene flow and drift [70], probably because expansions (both demographic and geographic) started from small effective population sizes and are relatively young, as revealed by the mismatch analyses. On the other hand, this conclusion should be considered with caution in light of the fact that we could cover only a small fraction of the eastern part of the species’ ranges.

Our results also pinpoint the difficulties in describing the population genetic structure in an ecotonal species, because of its simultaneous exposure to forces typical of two ecosystems (land and sea). The semi-terrestrial life style acquired by *O. montagui* prevents the species from realizing long-distance dispersal at sea at a rate comparable to that of truly marine organisms, especially those with a planktonic stage in their life cycle. The sea, on the other hand, functions as a means for passive dispersal by carrying individuals of *O. montagui* with floating materials across sites. The species’ genetic structure can indeed be largely explained in light of the pattern of circulation of surface sea currents. This passive process, being intrinsically unpredictable and variable, cannot counteract the onset of local genetic differences. Passive dispersal via floating material is extremely difficult to observe in nature and no information exists either on the rate of survival of these amphipods during the process or on their chances to settle on new sites following dispersal. Future avenues of research should foresee the development of ecological experiments capable of tracking single biological particles (talitrids in this case) in floating wracks to ultimately link genetic and oceanographic data in a unified framework [71].

## Competing interests

The authors declare that they have no competing interests.

## Authors’ contribution

LP collected the samples, designed the project, carried out the experiments and the statistical analyses, interpreted the data, drafted and critically revised the manuscript. VK collected the samples, designed the project, carried out part of the statistical analyses, interpreted the data and critically revised the manuscript. RT provided funding for all experiments and critically revised the manuscript. EDM provided the allozymic data and critically revised the manuscript. All authors read and approved the final manuscript.
